# Single‐Cell Sequencing Reveals the Immunosuppressive Trajectory in the Tumor Microenvironment of Human Giant Cell Tumor of Bone

**DOI:** 10.1155/bmri/9855803

**Published:** 2026-02-02

**Authors:** Yiming Liu, Wei Luo, Yude Xu, Xiguan Yao, Libing Dai, Qiao Feng, Peigeng Wang, Weichao Yang, Yi Feng, Haixiong Miao, Suixiang Huang, Dongping Ye

**Affiliations:** ^1^ Department of Orthopedics, Guangzhou Red Cross Hospital, Guangzhou Red Cross Hospital of Jinan University, Guangzhou, Guangdong, China, gzrch.com; ^2^ Department of Pain Medicine, Guangzhou Red Cross Hospital, Guangzhou Red Cross Hospital of Jinan University, Guangzhou, Guangdong, China, gzrch.com; ^3^ Guangzhou Institute of Traumatic Surgery, Guangzhou Red Cross Hospital, Guangzhou Red Cross Hospital of Jinan University, Guangzhou, Guangdong, China, gzrch.com; ^4^ Department of General Practice, The First Affiliated Hospital of Jinan University, Jinan University, Guangzhou, Guangdong, China, jnu.edu.cn

**Keywords:** giant cell tumor of bone, immunosuppression, single-cell sequencing, tumor-associated macrophages, tumor microenvironment

## Abstract

**Background:**

Giant cell tumor of bone (GCTB) presents considerable complexity in tumor microenvironment (TME) because of its intricate intercellular heterogeneity and the presence of an immunosuppressive milieu. In order to understand the complex gene expression patterns and cell interactions in GCTB, we carried out a thorough investigation using single‐cell RNA sequencing (scRNA‐seq).

**Methods:**

We examined scRNA‐seq data from 7091 cells that were collected after surgical removal of GCTB. Following the initial quality control process, 10 separate groups of cells were distinguished, which consisted of dendritic cells, endothelial cells, macrophage cells, mast cells, monocyte cells, neutrophil cells, tumor cells, osteoclast cells, pericyte cells, and T cells. Additional analysis uncovered distinct categories within tumor‐associated macrophages (TAMs), CD8+ T cells, and CD4+ T cells. The differentiation mechanisms of TAMs, CD8+ T cells, and CD4+ T cells were explored using pseudo‐time trajectory analysis. The CellPhoneDB study revealed the interactions between various cell types within the TME of GCTB.

**Results:**

TAMs have been identified as the main infiltrating cells in GCTB. These TAMs exhibit several subtypes that are characterized by specific marker genes and functional states. The identification of several subgroups within CD8+ T cells that are involved in regulating immunological checkpoints underscores the difficulties encountered when attempting to employ immune checkpoint blockade therapy for GCTB. T cell exhaustion poses a major barrier to the efficacy of antitumor immune responses. Research suggests a strong correlation between TAMs and exhausted T cells (Texs) in the TME. The high number of regulatory T cells (Tregs) highlights the immunosuppressive nature of the immunological environment in GCTB. Significant interactions have been observed between TAMs and tumor cells, highlighting their crucial involvement in immune evasion strategies.

**Conclusion:**

This scRNA‐seq study provides a general overview of the different cellular compositions and immune interactions within GCTB. The identified subtypes and communication networks provide valuable information about the immunosuppressive environment of GCTB, laying the foundation for prospective therapeutic approaches targeting specific cell types or interactions.

## 1. Introduction

Giant cell tumor of bone (GCTB), prevalent in the Asian population, is a type of bone tumor [1]. The condition primarily impacts persons in the younger age range, often between 20 and 40 years old, accounting for 60%–75% of occurrences [2]. Although GCTB is classified as an intermediate tumor, it displays significant local invasiveness and has a high likelihood of recurring and potentially transforming into a malignant form [3]. It exhibits a significant capacity for bone tissue degradation, locally invasive capabilities, elevated rates of recurrence, and a specific inclination for metastasis. From a histological perspective, this condition is distinguished by the existence of multinucleated large cells (hence the name) and mononuclear cells [4, 5]. Multinucleated giant cells display both functional and physical characteristics that are similar to those of osteoclasts [6–8]. Furthermore, mononuclear cells consist of proliferating spindle stromal cells (a constituent of tumors) and osteoclast precursors, which belong to the mononuclear macrophage lineage [9, 10]. GCTB presents as a condition where there is local invasion and bone resorption caused by osteoclasts. This typically results in the tumor coming back after it has been surgically removed [11, 12].

The emergence and growth of tumors are a complicated and multifaceted process influenced by different elements inside the intricate tumor ecology. The diversity of cancer cells interacts with both immune and nonimmune cells, giving rise to a complex tumor microenvironment (TME). Several studies have shown the vital role of the intricate and complicated TME in the initiation and progression of malignancies [13–15].

The utilization of single‐cell high‐throughput sequencing technology enables the identification of unique characteristics of individual cells, leading to a more comprehensive and precise depiction of disease conditions. This technique offers numerous benefits. The procedure entails the isolation of individual cells, the application of distinct labels to each cell, and the subsequent amplification and sequencing of the libraries. This method enables the tracking of individual cells, unveiling patterns of gene activation or repression in each cell, thereby carefully studying intercellular heterogeneity. It has a vital function in the early identification, monitoring, personalised treatment, and exploration of processes associated with the growth of multicellular organisms [16]. Presently, single‐cell transcriptome sequencing has been extensively utilized in several domains, such as tumor heterogeneity [17], immunological microenvironment [18], tissue injury and repair [19], reproductive development [20], brain growth [21], and cell differentiation [22].

By employing single‐cell transcriptome analysis, it is possible to comprehensively investigate immune cells within the complex TME. Researchers have recently employed scRNA‐seq to examine immune cells that have invaded tumors in several cancer types, including cutaneous melanoma [23, 24], non‐small cell lung cancer [25, 26], hepatocellular carcinoma [27], basal cell carcinoma [28], colorectal cancer [29, 30], and breast cancer [31]. These investigations have uncovered notable diversity in the immunological profile of tumors, separate subgroups of immune‐suppressive cells, undefined subcategories of immune cells, and specialized signaling networks that are unique to different forms of cancer. Nevertheless, GCTB has not yet undergone such analysis. This work utilized scRNA‐seq to investigate the TME of GCTB. We conducted a comprehensive investigation of the cellular makeup and gene expression patterns in the TME of GCTB utilizing single‐cell transcriptome analysis. Our study revealed a detailed comprehension of the immune‐suppressive traits of the TME at the cellular level. This allows for a more profound comprehension of the pathogenic mechanisms of GCTB and aids in the development of potential innovative therapeutic strategies.

## 2. Methods

### 2.1. Collection of Patients and Samples

The research received approval from Guangzhou Red Cross Hospital, which is connected with Jinan University, and adhered to all applicable ethical regulations. Prior to this trial, participants were given informed consent to provide their clinical and cell data. The study collected tissue samples of GCTB from a single patient who had “Resection of giant cell tumor of distal femur+left knee joint replacement surgery” at the Orthopedic Department of the Guangzhou Red Cross Hospital. Patients presenting with additional tumor tissue lesions were not included in the study, and the collected materials were later confirmed to include giant cell tumor tissue by additional postoperative pathological testing. The newly obtained samples obtained during the surgical procedure were conserved in MACS tissue storage solution (Miltenyi Biotec, Germany) and immediately sent to the laboratory.

### 2.2. Preparation of Single‐Cell Suspension

Following surgical tissue excision, the specimens were preserved in MACS tissue storage solution (Miltenyi Biotec) until they underwent processing. Initially, the materials were purified using phosphate‐buffered saline (PBS) and then broken down into small 1 mm^3^ fragments while kept on ice. Following that, enzymatic hydrolysis was carried out at a temperature of 37°C for a duration of 70 min, utilizing 3 mg/mL collagenase I (Worthington), 6 mg/mL collagenase II (Worthington), 4 mg/mL Dispase II (Sigma), and a 10% concentration of fetal bovine serum (FBS) in DMEM, with continuous stirring. Following the process of digestion, the materials were filtered using a cell strainer with a hole size of 70 *μ*m. The resulting cell suspension was then subjected to centrifugation at a force of 300 g for a duration of 5 min. Following the removal of the liquid portion containing suspended particles, the cluster of cells was reconstituted in a specialized solution called red blood cell lysis buffer (Miltenyi Biotec) in order to rupture and dissolve the red blood cells. After washing with a PBS solution containing 0.04% BSA, the cell clumps were resuspended in another PBS solution containing 0.04% BSA and then filtered through a 35‐*μ*m cell strainer. Afterwards, individual cells were stained with Am/Draq7 for the purpose of separation, and the BD LSRFortessa cell analyzer was used to determine their vitality.

### 2.3. Library Construction and scRNA‐Seq Using a Pre‐Prepared Suspension of Individual Cells

The BD Rhapsody Analysis System was employed for the purpose of performing single‐cell whole‐transcriptome sequencing. The cell suspension was introduced inside BD Rhapsody cartridges that included microfluidic arrays, each with partitions exceeding 220,000 in number. The process of capturing individual cells was accomplished by enabling the random placement of single cells to be immobilized by the force of gravity within the microwells. Oligonucleotide‐labeled magnetic beads were introduced to bind with cells. Following the lysis of cells, the mRNA was captured using barcode magnetic beads, which were then retrieved, washed, and treated with reverse transcription and RNase I digestion [32]. The process of preparing single‐cell transcriptome sequencing libraries involved several PCR processes, performing random priming and extension (RPE), RPE PCR, and whole‐transcriptome amplification (WTA) index PCR according to the manufacturer′s instructions. The standardised libraries were subjected to sequencing on the NovaSeq Illumina platform utilizing 150 bp paired‐end reads.

### 2.4. Analyzing Data From scRNA‐Seq

The processing of the scRNA‐seq data was carried out using the NovelBrain cloud computing platform. The fastp approach was employed to eliminate adaptor sequences, discard low‐quality reads, and purify the data, utilizing the default parameters. The UMI‐tools were employed to analyze single‐cell transcriptomes to identify whitelists of cell barcodes. The clean data, based on unique molecular identifiers (UMIs), was aligned to the human genome (GRCh38 Ensemble: Version 104) using the STAR mapping algorithm. The mapping process was customised by using parameters from the UMI‐tools standard workflow. Through this mapping method, we were able to obtain UMI numbers for each individual sample. Cells exhibiting over 200 expressed genes, mitochondrial UMI rates below 20%, and high‐quality were excluded. The expression matrix was depleted of mitochondrial genes [33].

### 2.5. Analysis, Visualization, and Annotation of Cell Clustering

The Seurat software (Version: 4.0.3, https://satijalab.org/seurat/) was used to standardise and adjust cells using the expression matrix, considering UMI counts and mitochondrial percentage. Principal component analysis (PCA) was performed using the scaled data of the Top 2000 genes that demonstrate significant variability. The tSNE and UMAP algorithms were applied using the Top 10 principal components. The fastMNN function from the scran package (v1.12.1) was employed to mitigate batch effects among samples. The parameters used were *k* = 5, *d* = 50, and approximation = TRUE. Cell clustering was conducted by utilizing a graph‐based clustering technique with a resolution of 0.8, employing the Top 10 PCA components. The identification of important genes was conducted using the FindAllMarkers function and the Wilcoxon rank‐sum test method, taking into account the specified parameters (1) the natural logarithm of FC is greater than 0.25; (2) The *p* value is less than 0.05, specifically (3) the minimum percentage is more than 0.1. To achieve precise cell type identification, We performed re‐tSNE analysis, graph‐based clustering, and marker analysis on clusters composed of cells belonging to the same type [34].

### 2.6. Pseudo‐Time Analysis

The Monocle 2 program was employed to analyze cellular trajectories at the individual cell level. Before conducting Monocle analysis, we chose the genes that were determined to be significant based on the Seurat clustering results, together with the raw expression counts of the filtered cells. The research of genes that regulate branching was conducted by utilizing pseudo‐time analysis and branch expression analysis modeling (BEAM analysis).

### 2.7. Cell Communication Analysis

In order to methodically examine molecules involved in cell–cell communication, we employed CellPhoneDB [35], an openly accessible database that encompasses data on ligands, receptors, and their interactions. Clusters of membrane, secretory, and peripheral proteins were detected at different time intervals. The statistical significance of the mean and cell communication (*p* value < 0.05) was calculated using the interactions and normalised cell matrices acquired by Seurat.

### 2.8. Single‐Cell Regulatory Network Inference and Clustering Analysis (SCENIC)

SCENIC is a software tool utilized for inferring gene regulatory networks and the associated cell states from single‐cell data. The analysis utilized transcription factor target databases, also known as transcription factor motif databases, to determine the expression of transcription factors and their target genes in the specific cell population. It then computed the regulatory genes and their regulatory strength, measured by the AUCell score, for each individual cell. By conducting this investigation, we have determined the regulation of transcription factors in various cell populations, which may help us find distinct transcription factors for each cell cluster. The SCENIC methodology, including pySCENIC (v0.9.5) [36], was employed to evaluate the regulatory potency of transcription factors.

### 2.9. QuSAGE Analysis

QuSAGE analysis, or gene set expression activation quantification analysis, is a method for quantitatively analyzing gene enrichment using gene sets and gene expression data. The variance inflation factor algorithm is used to perform GSEA‐like enrichment analysis of gene sets. By employing this approach, gene set enrichment analysis was effortlessly performed on various clusters, including the Kyoto Encyclopedia of Genes and Genomes (KEGG) gene sets, GSEA gene sets, and even gene sets curated by academics, to assess the disparities in enrichment across different clusters. In order to assess the level of activation of a specific collection of genes and investigate pathway activation, we performed a study utilizing QuSAGE (2.16.1) [37].

### 2.10. Gene Ontology (GO) Analysis

GO analysis was performed to elucidate the biological significance of marker genes and genes exhibiting differential expression. The GO annotations were obtained from the NCBI, UniProt, and GO websites. The Fisher′s exact test was employed to detect significant GO categories, and the *p* values were adjusted using the false discovery rate (FDR) technique.

## 3. Results

### 3.1. scRNA‐Seq Reveals the Cellular Composition of GCTB

scRNA‐seq study was performed on samples from individuals who had been diagnosed with GCTB after surgical excision of the tumor (Figure 1a). Following an initial quality control assessment, we utilized scRNA‐seq data from 7091 cells derived from primary cells for subsequent analysis. The distribution following the reduction of dimensions revealed that cells in close proximity displayed a higher level of resemblance in their genetic expression patterns. After doing an initial quality control review, we used scRNA‐seq data from 7091 cells that were obtained from primary cells for further study. The distribution following the reduction of dimensions revealed that cells in closer proximity displayed a higher level of similarity in their patterns of gene expression. A total of 10 prominent cell clusters were found (Figure 1b) (1) dendritic cells, (2) endothelial cells, (3) macrophage cells, (4) mast cells, (5) monocyte cells, (6) neutrophil cells, (7) tumor cells, (8) osteoclast cells, (9) pericyte cells, and (10) T cell cells.We performed a comparison study to determine the proportions of each cell category in the samples. Significantly, the samples exhibited the greatest proportion of macrophage cells, followed by T cells, tumor cells, osteoclast cells, and mononuclear cells (Figure 1c). The cell populations found exhibited similarities to prior investigations, indicating that GCTB is distinguished by a notable abundance of osteoclast‐like large cells and closely packed clusters of mononuclear cells [38]. The predominant immune cells found in GCTB are macrophages and T lymphocytes. The characteristic indicators for each cellular cluster are as follows (1) the observed cell types include dendritic cells (DC) (FCER1A), endothelial cells (CDH5 and FLT1), macrophage cells (MSR1 and CD86), mast cells (TPSAB1 and CPA3), monocyte cells (CCR2), neutrophil cells (S100A9), tumor cells (RANKL, RUNX2, and IBSP), osteoclast cells (CTSK and ACP5), pericyte cells (RGS5.1 and PDGFRB), and T cells (IL7R, CD3D, and CD3E) (Figure 1d).

Figure 1ScRNA‐seq profile of 7091 cells from human samples of GCTB. (a) Workflow depicting the collection and processing of GCTB tumor specimens for scRNA‐seq. (b) Based on uniform manifold approximation and projection (UMAP), dimensionality reduction visualization is performed and coloring is marked according to cell type, from 7091 cells of GCTB sample. (c) Proportional Pie Chart representing the proportions of 10 different cell types in GCTB samples. (d) The UMAP plots display the expression of well‐known markers across different cell types in GCTB.

**Figure:**
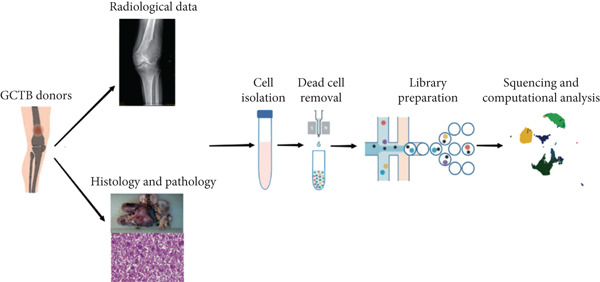
(a)

**Figure:**
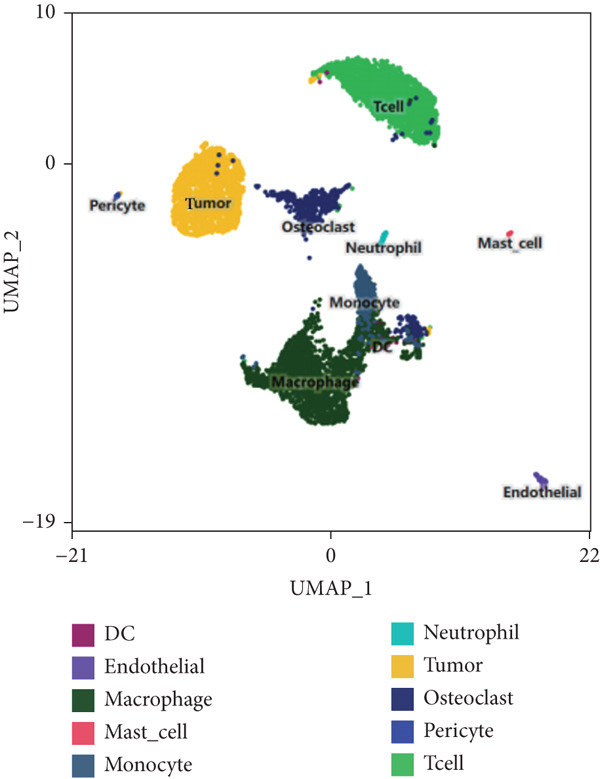
(b)

**Figure:**
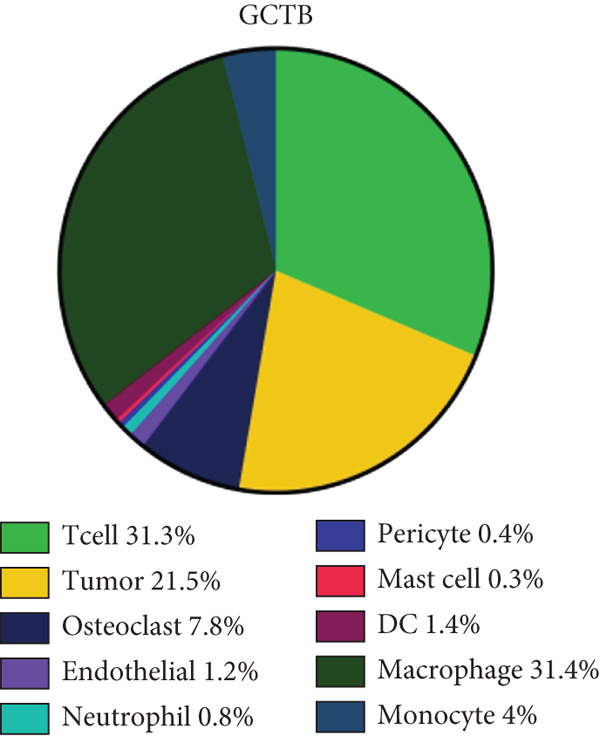
(c)

**Figure:**
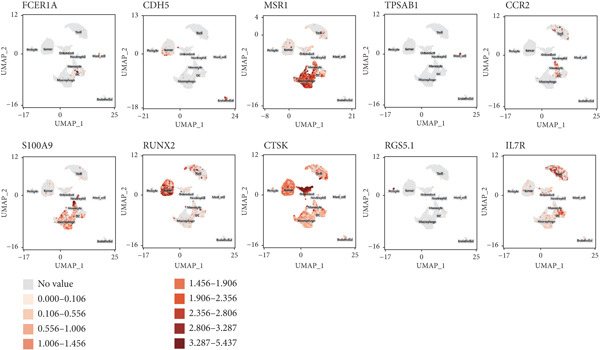
(d)

#### 3.1.1. The Variability of Tumor‐Associated Macrophages (TAMs) in GCTB

Reclustering of 2226 macrophages yielded the discovery of 10 different subgroups (Figure 2a). A heat map was created using the top 20 marker genes that showed the highest differential selection (Figure 2b). We identified and characterized different subtypes of macrophages using unbiased clustering, literature investigation, and databases. Each subtype was associated with specific marker genes. The macrophage subtypes and their corresponding marker genes are as follows: Macro‐C1 (EGR1 and KLF2), Macro‐C2 (SPP1, HMOX1, and CCL18), Macro‐C3 (C1QC and FOLR2), Macro‐C4 (FCN1, TREM1, and MARCO), Macro‐C5 (HLA‐DRA), Macro‐C6 (IL7R, and FN1), Macro‐C7 (NEAT1), Macro‐C8 (CXCL9, CXCL10, and ISG15), Macro‐C9 (MKI67, and TOP2A), and Macro‐C10 (IL1RN, CSTB, and LGALS3) (Figure 2c).

Figure 2ScRNA‐seq profile of 2226 TAMs from human samples of GCTB. (a) UMAP plot of 2226 cells demonstrating the 10 main cell types in TAMs. (b) Heat map showing and highlighting differentially expressed genes for each cluster of TAMs. (c) UMAP plots showing the expression of representative well‐known markers for each cell type in TAMs. (d) Bubble plots illustrate the expression levels of marker genes in 10 cell clusters. The color of each point indicates the percentage of gene expression within the respective cell cluster, whereas the size of the point corresponds to the percentage of gene expression in the cell cluster. (e) Proportional Pie Chart representing the proportions of 10 different cell types in TAMs samples.

**Figure:**
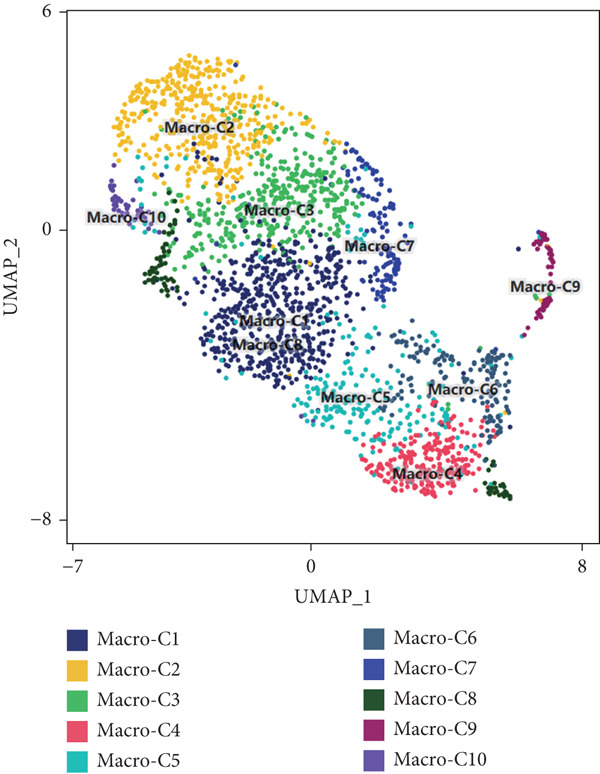
(a)

**Figure:**
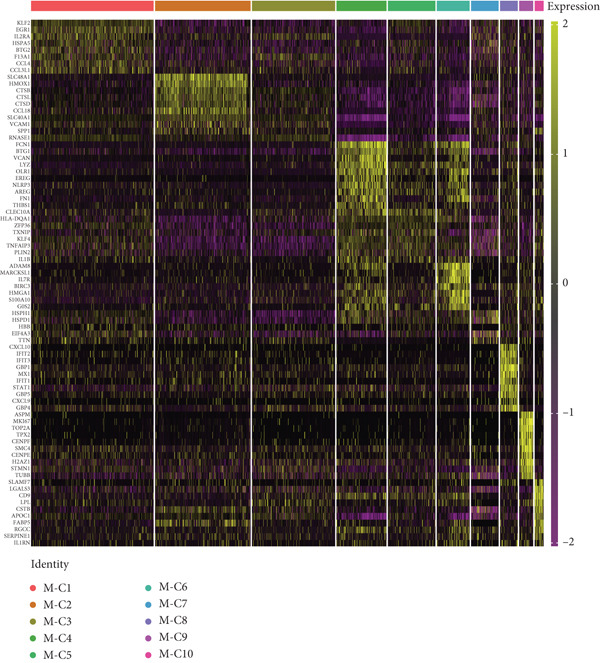
(b)

**Figure:**
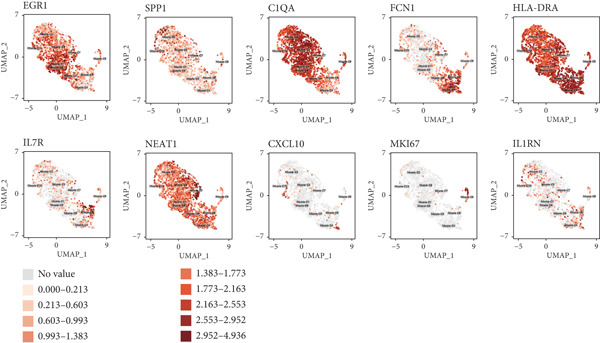
(c)

**Figure:**
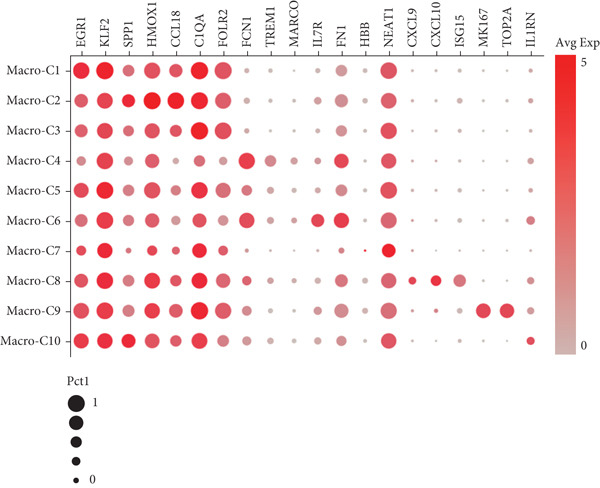
(d)

**Figure:**
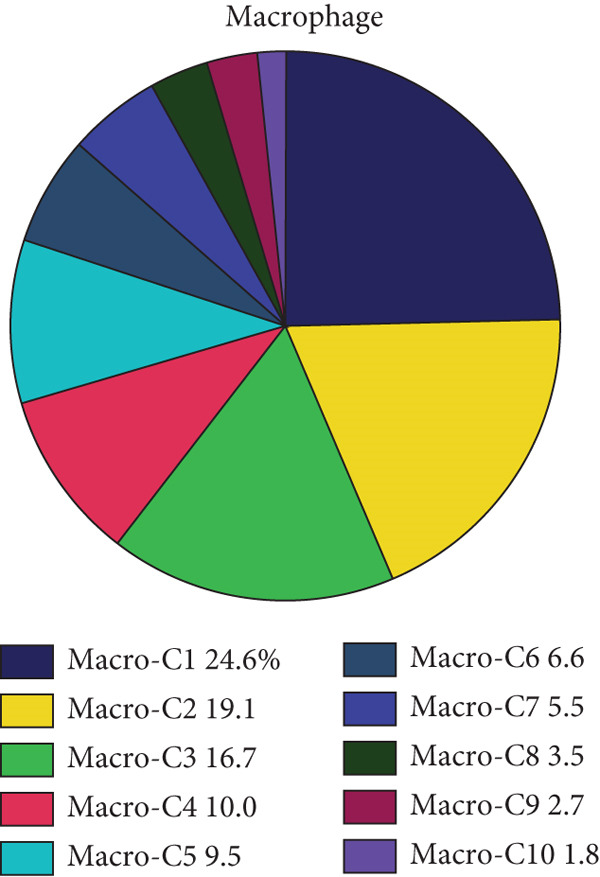
(e)

We employed bubble plots to visualise the collective expression levels of these marker genes across the 10 cell clusters (Figure 2d). Macro‐C1 demonstrated increased expression levels of EGR1, a protein known to stimulate the growth of blood vessels in tumors [39]. The expression levels of SPP1, HMOX1, and CCL18 were found to be high in Macro‐C2, suggesting that it belongs to the SPP1‐polarized TAM subtype. This subtype is known to be associated with hypoxic settings and plays a role in promoting tumor angiogenesis. Moreover, Macro‐C8 exhibited elevated levels of CXCL9, CXCL10, and ISG15, so classifying it as the CXCL9‐polarized TAM subtype. Prior research indicated a mutually exclusive association between Macro‐C2 and Macro‐C8, in which SPP1‐high TAMs exhibit pro‐tumoral properties, whereas elevated CXCL9 expression indicates anti‐tumoral traits [40]. Tissue‐resident macrophages were identified as Macro‐C3, which is distinguished by its high expression of C1QC and FOLR2. The presence of a high number of FOLR2 + macrophages showed a direct relationship with improved patient survival rates, suggesting that these macrophages have anti‐tumoral properties [41]. Macro‐C4, which produces FCN1, TREM1, and MARCO, indicates an intermediary stage between monocytes and fully developed macrophages, commonly referred to as myeloid‐derived suppressor cell‐like TAMs. Prior research has shown that these immature macrophages are more abundant in the TME when there is a dearth of CD8[+] T cells. This association is linked to behaviors that promote tumor growth [42, 43]. Macro‐C5 demonstrated elevated expression of HLA‐related genes, whereas Macro‐C6 displayed significant expression of IL7R, showing traits associated with residence. Macro‐C7 exhibited elevated expression levels of NEAT1, whereas Macro‐C9 demonstrated increased expression of MKI67 and TOP2A, suggesting their proliferative tumor‐associated macrophage characteristics. Macro‐C10, which contains IL1RN, CSTB, and LGALS3, appears to have a role that may be associated with anti‐inflammatory effects. The majority of TAMs are accounted for by Macro‐C1, Macro‐C2, and Macro‐C4, which actively promote the tumor phenotype (Figure 2e).

#### 3.1.2. Analysis of the Functions and Status of Various Subtypes of TAMs in GCTB

By conducting a more in‐depth examination of the sequencing data, we conducted GO enrichment analysis to classify the significantly different genes in TAMs into specific functional categories. This process revealed separate biological processes (BP) for various TAM subtypes: Macro‐C1—inflammatory response, response to unfolded protein, and negative regulation of apoptotic processes. Macro‐C2—neutrophil degranulation, transferrin transport, and lipid metabolic processes. Macro‐C3—neutrophil degranulation, viral entry into host cells, and inflammatory responses. Macro‐C4—SRP‐dependent cotranslational membrane targeting, cytoplasmic translation, and translation initiation. Macro‐C5—cytoplasmic translation, viral transcription, and antigen processing and presentation. Macro‐C6—cytoplasmic translation, messenger RNA degradation processes, and viral transcription. Macro‐C7—positive regulation of RNA polymerase II transcription, RNA splicing, and mRNA processing. Macro‐C8—defense response to viruses, Type I interferon, and inflammatory responses. Macro‐C9—cell cycle, cell division, and DNA replication. Macro‐C10—long‐chain fatty acid transport and lipid metabolic processes (Figure 3a).

Figure 3Characteristics of TAMs in GCTB (a) The bar chart illustrates the gene ontology (GO) biological process analysis of 10 cell clusters of TAMs. (b) The bar chart presents the Kyoto Encyclopedia of Genes and Genomes (KEGG) enrichment analysis of 10 cell clusters of TAMs. (c) The QuSAGE heat map displays the gene sets enriched in the 10 cell clusters of TAMs. (d). Single‐cell regulatory network inference and clustering (SCENIC) analysis revealed genes associated with transcriptional regulatory networks in different cell clusters of TAMs.

**Figure:**
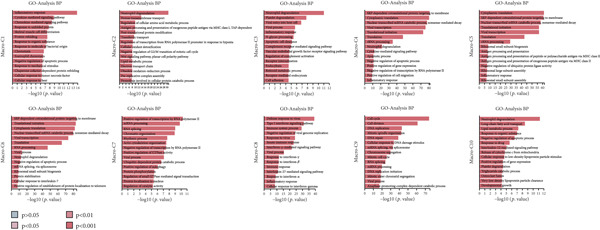
(a)

**Figure:**
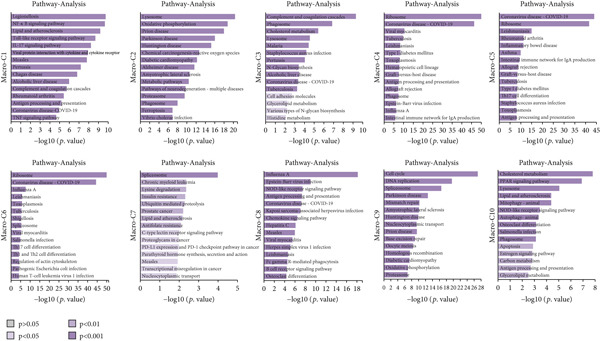
(b)

**Figure:**
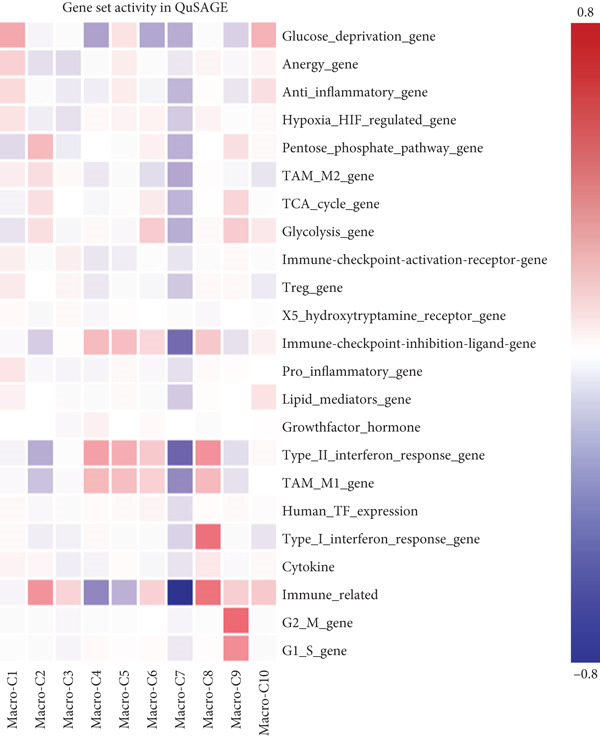
(c)

**Figure:**
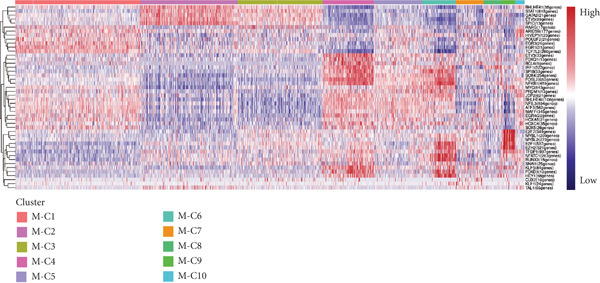
(d)

Furthermore, The KEGG enrichment study revealed varying degrees of enhanced signaling pathway expressions across distinct cell groups: Macro‐C1—NF‐*κ*B signaling pathway, lipid and atherosclerosis, and toll‐like receptor signaling pathway. Macro‐C2—lysosome, oxidative phosphorylation, ferroptosis. Macro‐C3—complement and coagulation cascades, phagosome, cholesterol metabolism. Macro‐C4—ribosome, viral myocarditis. Macro‐C5—coronavirus disease (COVID‐19) and rheumatoid arthritis. Macro‐C6—ribosome, Th17 cell differentiation, and Th1 and Th2 cell differentiation. Macro‐C7—spliceosome, chronic myeloid leukemia, lysine degradation. Macro‐C8—epstein‐Barr virus infection, NOD‐like receptor signaling pathway, and osteoclast differentiation. Macro‐C9—cell cycle, DNA replication, and spliceosome. Macro‐C10—cholesterol metabolism and PPAR signaling pathway (Figure 3b).

Furthermore, the QuSAGE gene set analysis approach was employed to detect gene sets that exhibited high enrichment in each subtype. Our observation revealed a high enrichment of the glucose deprivation gene set in Macro‐C1 and Macro‐C10, suggesting an increased level of metabolic activity. Macro‐C2 displayed a detrimental correlation with TAM M2 genes and Type II interferon response genes, indicating an anti‐inflammatory characteristic. In contrast, Macro‐C8 exhibited a contrasting trend in these gene sets, with a significant enrichment of Type I Interferon response genes, which suggests an intensified immune response activity. The proliferative activity of Macro‐C9 was emphasized by the active expression of G2 M genes and G1 S genes (Figure 3c).

To summarize, our analysis offers a thorough comprehension of the functional variety and conditions of several TAM subtypes in GCTB. The SCENIC analysis uncovers transcriptional regulatory networks linked to distinct cell types: Macro‐C1—EGR1 and EGR3. Macro‐C2—SPIC and GATA2. Macro‐C3—RARG. Macro‐C4—BCL6 and FOXQ1. Macro‐C5—HOXA5. Macro‐C6—RUNX3 and NFATC1. Macro‐C7—CUX2 and KLF1. Macro‐C8—IRF1. Macro‐C9—E2F7 and MYBL1. Macro‐C10—HIVEP1 (Figure 3d).

#### 3.1.3. TAMs Differentiation Process in GCTB

In order to study the process of differentiating of TAMs and the genes that are expressed during this process, we created a pseudo‐temporal trajectory using 10 different cellular subtypes (Figure 4a). The trajectory displays the distribution of 10 subtypes of TAMs in various clusters along the pseudo‐temporal axis. It also shows the arrangement of clusters based on their proximity in pseudo‐temporal space, as well as the overall distribution of inferred virtual state statuses using pseudo‐temporal analysis. When analyzing clusters separately, with Node 2 as a border, we noticed that the initial cell state consisted of proliferative TAMs of the Macro‐C9 subtype, which then differentiated into the Macro‐C2 subtype characterized by SPP1 polarization. The population in Fate2 was mainly composed of Macro‐C1 and Macro‐C7. The Macro‐C8 subtype, which is polarized by CXCL9, is more abundant at the mid‐differentiation stage and becomes more prevalent in the later stages of Fate1. Toward the end of Fate1, there is a significant increase in the Macro‐C4 subtype, which resembles marrow‐derived suppressor cells.

Figure 4Molecular profile of TAMs during GCTB initiation and progression by scRNA‐seq. (a) The Monocle trajectory analysis plot illustrates the progression of differentiation for the 10 clusters of TAMs in GCTB. (b) The heat map illustrates the progression of enriched gene sets from the prestate to Fate1 and Fate2 in the trajectory analysis. (c) This bar chart illustrates the gene ontology (GO) biological process analysis of three different modules. (d) The bar chart presents the Kyoto Encyclopedia of Genes and Genomes (KEGG) enrichment analysis of three different modules.

**Figure:**
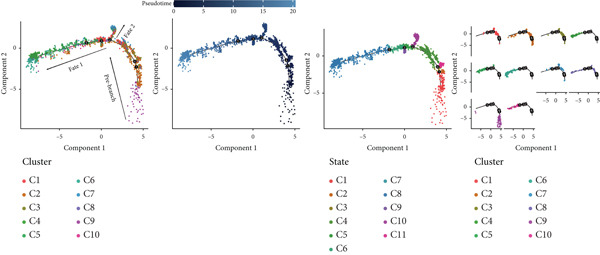
(a)

**Figure:**
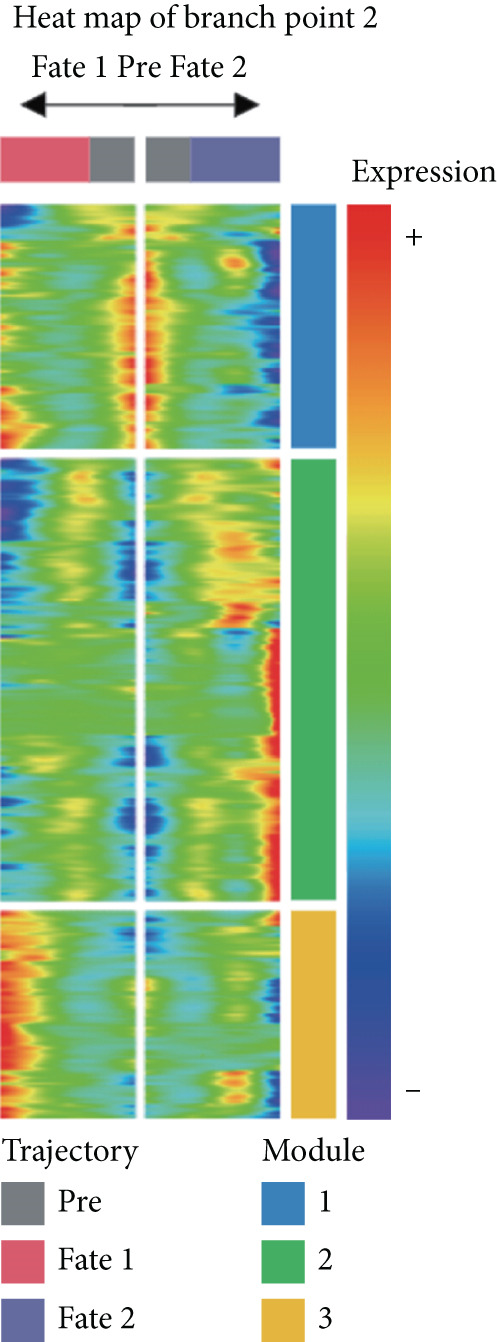
(b)

**Figure:**
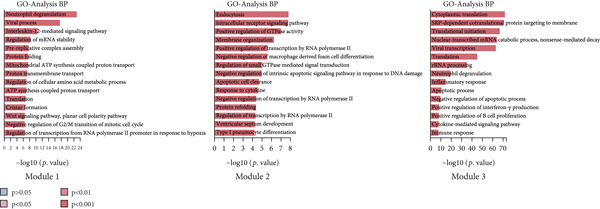
(c)

**Figure:**
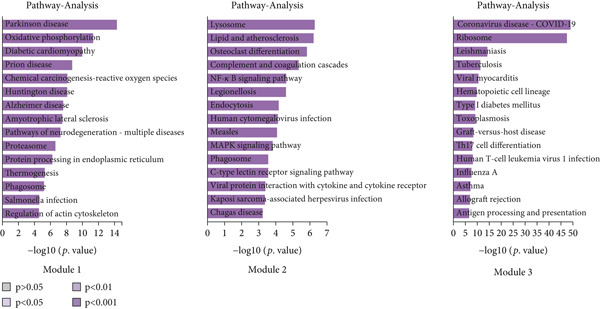
(d)

To further investigate the specific biological roles of these varied outcomes, a heat map of branch point was created for TAMs (Figure 4b). It unveiled three modules that depict the process of macrophage differentiation. Additional GO BP enrichment analysis (Figure 4c) and pathway analysis (Figure 4d) were performed on these modules. Module 1, which corresponds to the starting trajectory, demonstrated greater differentiation and proliferative capacity. Module 2, which corresponds to Fate2, potentially regulates the process of osteoclast differentiation. On the other hand, module 3, corresponding to Fate1, is primarily linked to immunological and inflammatory response functions.

#### 3.1.4. Variability of CD8+ T Cells in GCTB

We have classified 951 reclustered CD8+ T cells into five distinct categories (Figure 5a). A heat map is shown, presenting the Top 20 marker genes that have been elevated. These genes were identified via differential screening (Figure 5b). By doing an impartial clustering analysis of the data and conducting thorough research in literature and databases, we have identified specific types of CD8+ T cells and their corresponding marker genes. These types are as follows (Figure 5c): C1 (HSPA1B and HSPA1A), C2 (CRTAM and GZMH), C3 (LAG3, HAVCR2, and CXCL13), C4 (IL7R and KLRB1), and C5 (MKI67 and TOP2A). In addition, we employed a bubble plot to illustrate the total expression levels of the respective marker genes for the five cell groupings (Figure 5d).

Figure 5ScRNA‐seq profile of 951 CD8+ T cells from human samples of GCTB. (a) UMAP plot of 951 cells demonstrating the five main cell types in CD8+ T cells. (b) Heat map showing and highlighting differentially expressed genes for five clusters of CD8+ T cells. (c) UMAP plots showing the expression of representative well‐known markers for each cell type in CD8+ T cells. (d) Bubble plots illustrate the expression levels of marker genes in five cell clusters. (e) Proportional pie chart representing the proportions of five different cell types in CD8+ T cells samples.

**Figure:**
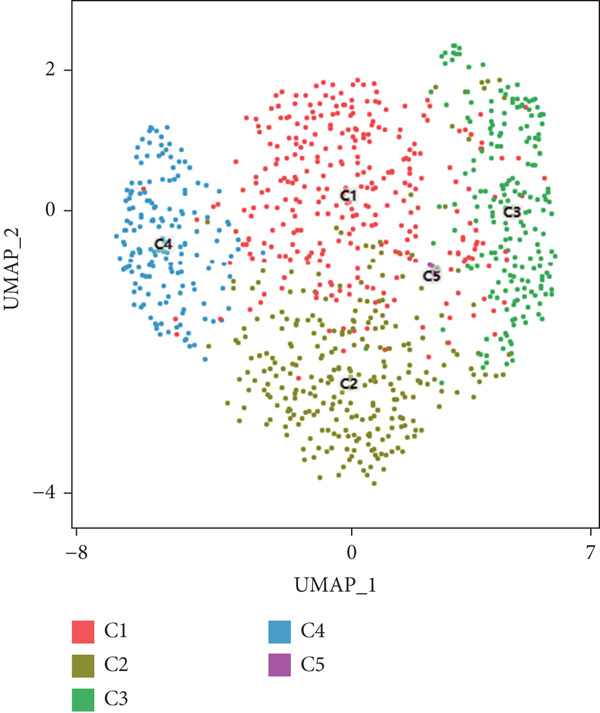
(a)

**Figure:**
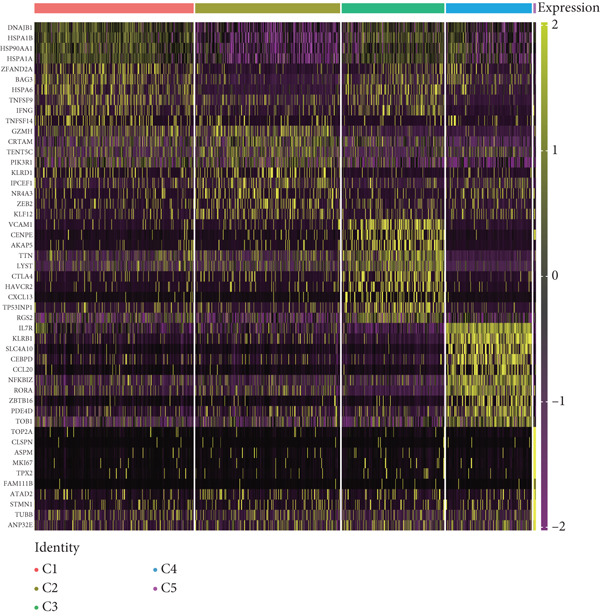
(b)

**Figure:**
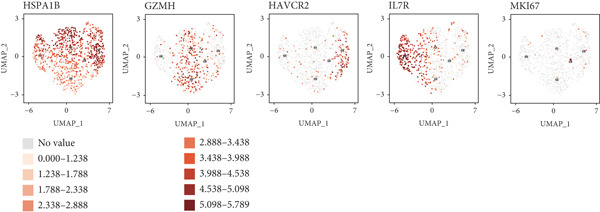
(c)

**Figure:**
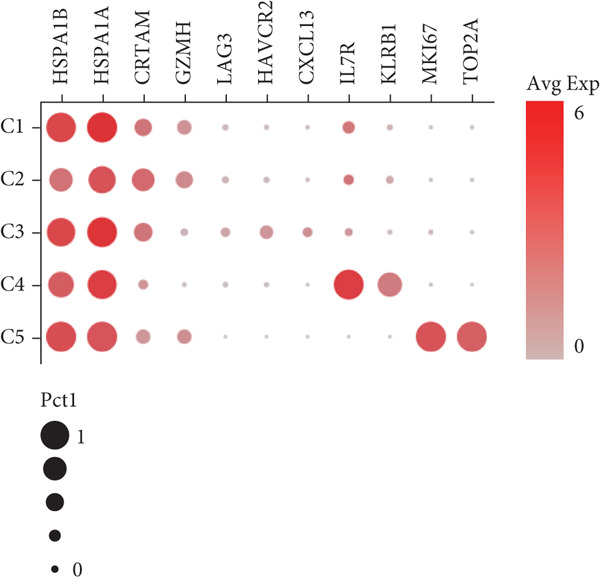
(d)

**Figure:**
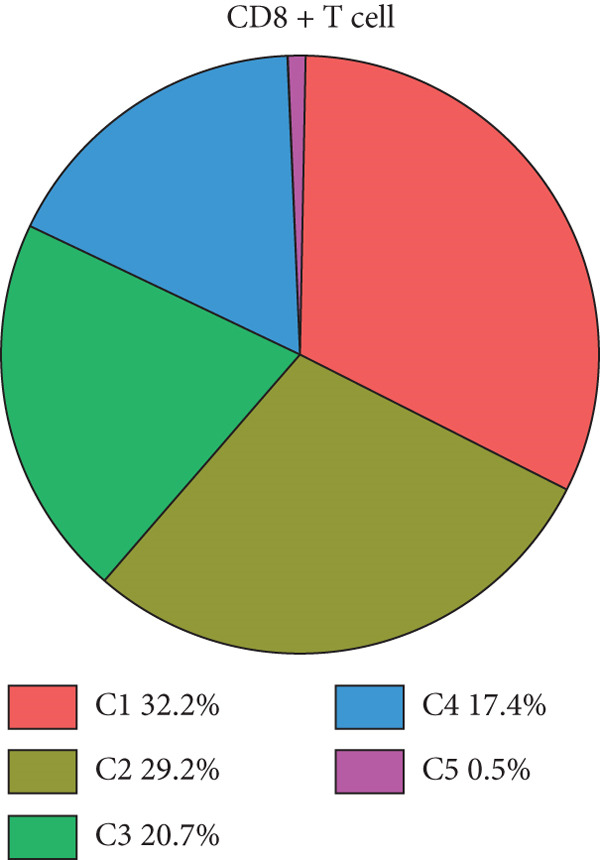
(e)

C1 exhibited an upregulation of stress‐related genes, including heat shock protein genes, indicating the presence of CD8+ T cell stress response state (TSTR). Tumor‐specific T cells with exhausted phenotypes, referred to as TSTR cells, are frequently observed in the tumors of patients who do not respond to immune checkpoint blockade therapy. This indicates that TSTR cells have a crucial function role in the resistance to immunotherapy [44]. C2, distinguished by elevated levels of CRTAM and GZMH expression, was classified as a cytotoxic subgroup. C3, characterized by elevated levels of LAG3, CXCL13, and HAVCR2, and containing a large proportion of genes associated to immunological checkpoints, signifies a state of exhaustion in CD8+ T cells [45]. Significantly, the genes IL7R, KLRB1, and CCL20 exhibited a significant level of activity in C4. Prior research has linked KLRB1, which encodes the CD161 protein, to the suppression of NK cell‐mediated cytotoxicity. Additionally, CD161 has been identified as a new immunological checkpoint on T cells [46]. The elevated concentration of CCL20 has been associated with the proliferation of tumors [47]. C5 exhibited significant enrichment of MKI67 and TOP2A, suggesting a high level of proliferative activity. An abundance of genes associated to immunological checkpoints and TSTR cells was seen within CD8+ T cells, representing a substantial proportion in GCTB (Figure 5e). This indicates a suppressed condition of the immune response against tumors in CD8 + T cells in GCTB.

#### 3.1.5. Functional and Status Analysis of CD8+ T Cell Subgroups in GCTB

We performed GO enrichment analysis on CD8+ T cells sequencing data, categorizing significantly different genes into specific functional categories within CD8+ T cells subgroups. The BP identified for each subgroup are as follows: C1—cellular response to heat and protein folding; C2—response to hypoxia, regulation of protein stability and pathway‐restricted SMAD prote; C3—negative regulation of T cell activation and intracellular signal transduction; C4—viral transcription and negative regulation of ubiquitin protein ligase activity; C5—cell cycle and DNA replication (Figure 6a).

Figure 6Characteristics of CD8 + T cells in GCTB. (a) The bar chart illustrates the gene ontology (GO) biological process analysis of five cell clusters of CD8 + T cells. (b) The bar chart presents the Kyoto Encyclopedia of Genes and Genomes (KEGG) enrichment analysis of five cell clusters of CD8+ T cells. (c) The QuSAGE heat map illustrates the gene sets enriched in the five cell clusters of CD8 + T cells. (d) Single‐cell regulatory network inference and clustering (SCENIC) analysis revealed genes associated with transcriptional regulatory networks in different cell clusters of CD8+ T cells.

**Figure:**
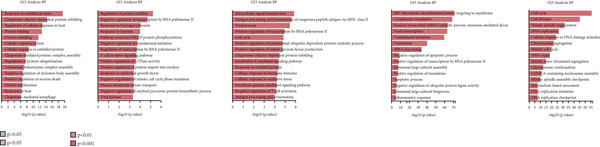
(a)

**Figure:**
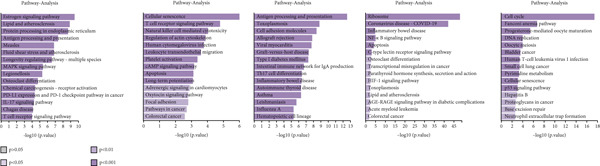
(b)

**Figure:**
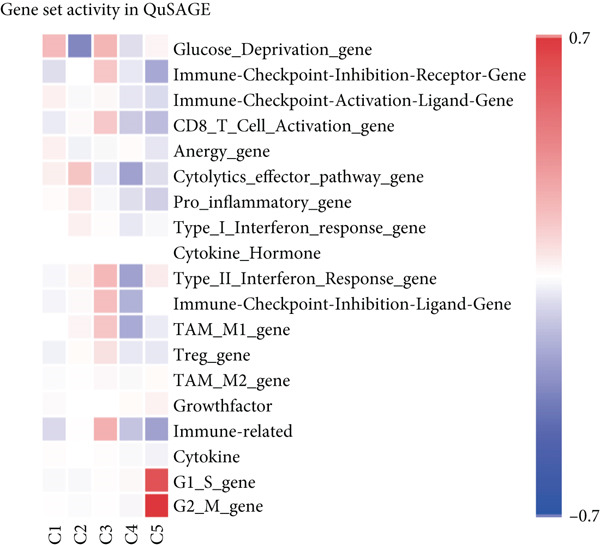
(c)

**Figure:**
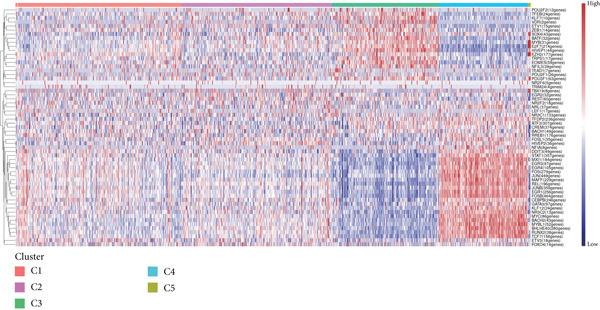
(d)

The KEGG enrichment analysis revealed distinct signaling pathways that exhibited variable enrichment across various cell groupings. In cell group C1, the enriched pathways were the estrogen signaling pathway, PD‐L1 expression and PD‐1 checkpoint, and lipid and atherosclerosis. In cell group C2, the enriched pathways were the T cell receptor signaling pathway and natural killer cell‐mediated cytotoxicity. In cell group C3, the enriched pathways were cell adhesion molecules, allograft rejection, and antigen processing and presentation. In cell group C4, the enriched pathways were the ribosome and coronavirus disease—COVID‐19. In cell group C5, the enriched pathways were the cell cycle and DNA replication (Figure 6b).

The QuSAGE gene set analysis found the gene sets that exhibited enrichment in the five subgroups of CD8+ T cells. Our observation revealed a higher enrichment of genes associated with improved metabolism, particularly glucose deprivation genes, in clusters C1 and C3. C2 demonstrated an increase in genes related to the cytolytic effector pathway, pro‐inflammatory response, and Type I interferon response, indicating its ability to cause cell death. In addition, C3 exhibited an increase in immune checkpoint genes, whereas G2 M genes and G1 S genes revealed a high level of proliferative activity in C5 (Figure 6c).

In addition, the analysis of SCENIC revealed specific genes connected to transcriptional regulatory networks for different subgroups of CD8+ T cells (Figure 6d): C1—ETV3; C2—TRIM24 and EGR2; C3—VDR and NFIL3; C4—NFIA and MYBL1; C5—SOX4 and EZH2.

#### 3.1.6. The Process of Differentiating CD8+ T Cells in GCTB

To further explore the process of differentiation in CD8+ T cells and the expression of their related gene, we created a pseudo‐temporal trajectory using five subgroups of cells (Figure 7a). The diagram depicts the distribution, temporal organization, and overall condition of virtual states inferred via pseudo‐temporal analysis for CD8+ T cell clusters, moving from left to right. Based on Node 1 as a reference point, the analysis identified the initial cell state as C4, which later transformed into C1 (stress‐like CD8+ T cells) and C2 cytotoxic cells (CRTAM and gzmh). A specific lineage, referred to as Fate1, exhibits a high concentration of genes associated with cell proliferation, but is characterized by a low presence of C5 cells. This lineage transitions into C3 cells, which are fatigued CD8+ T cells, toward the end of Fate2. This indicates the progressive depletion of CD8+ T cells during the differentiation process in GCTB. This aligns with our previous examination of TAMs in GCTB.

Figure 7Molecular profile of CD8+ T cells during GCTB initiation and progression by scRNA‐seq. (a) The Monocle trajectory analysis plot illustrates the progression of differentiation for the five clusters of CD8+ T cells in GCTB. (b) The heat map illustrates the progression of enriched gene sets from the prestate to Fate1 and Fate2 in the trajectory analysis. (c) The bar chart illustrates the gene ontology (GO) biological process analysis of three different modules. (d) The bar chart presents the Kyoto Encyclopedia of Genes and Genomes (KEGG) enrichment analysis of three different modules.

**Figure:**
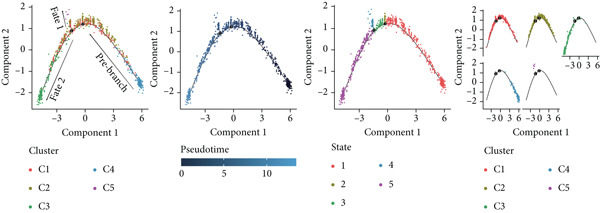
(a)

**Figure:**
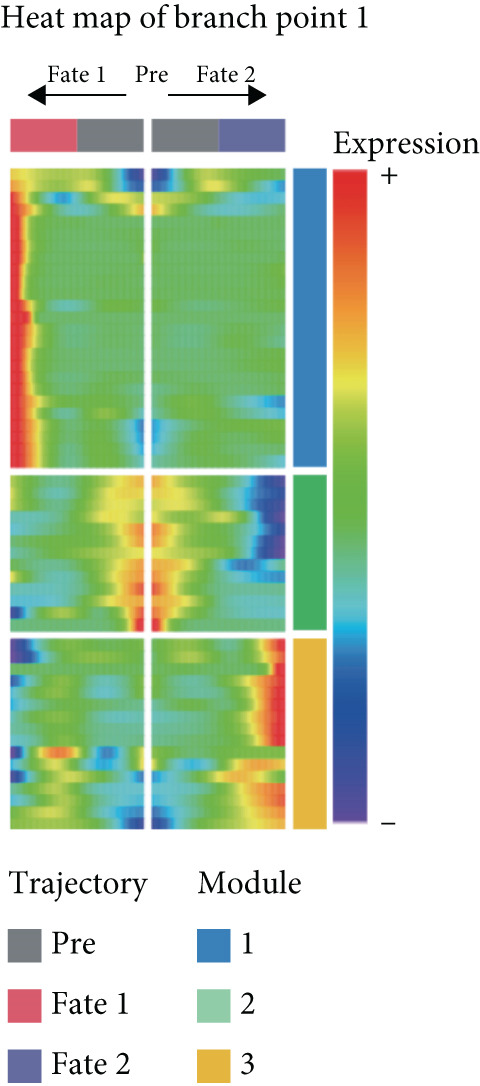
(b)

**Figure:**
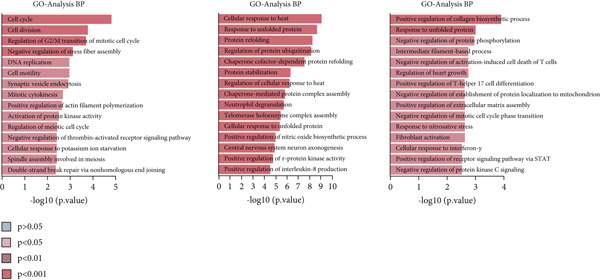
(c)

**Figure:**
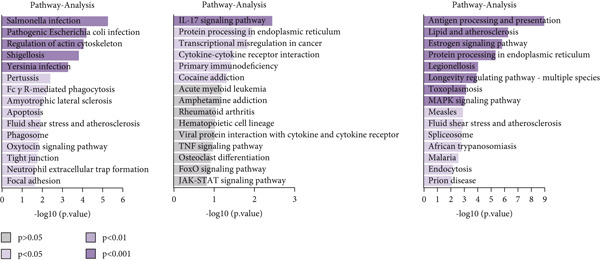
(d)

To conduct an investigation the main biological functions of various outcomes, we generated a heat map of branch point for CD8+ T cells, dividing them at Node 1 (Figure 7b). This heat map depicts three distinct modules of CD8+ T cells. Performing GO biological process enrichment analysis (Figure 7c) and pathway analysis (Figure 7d) on these modules revealed the following associations: Module 1 corresponds to Fate1 and exhibits a stronger proliferative potential; Module 2 represents the initial characteristics of resident CD8+ T cells [48]; and Module 3 signifies the highly enriched exhausted state of CD8+ T cells in Fate2.

#### 3.1.7. The Multiplex Immunofluorescence Staining

To verify the presence of PDCD 1 CD8+ T cells in the GCTB tumor tissue, we performed multiplex immunofluorescence staining of the samples, and the results confirmed the presence of CD8 expressing PDCD 1 in the tumor samples + T cell.

Paraffin sections were placed in xylene I for 15 min, with absolute ethanol I, absolute ethanol, 85% alcohol, and 75% alcohol for 5 min each, and then washed with distilled water. Sections were placed inside the repair box and subjected to antigen repair at high temperatures. After natural cooling, it was placed in PBS (pH 7.4) and washed for 5 min for three times. The sections were incubated in 3% hydrogen peroxide (25 min) and placed in PBS (pH 7.4) and subsequently washed for 5 min in a decolorization shaker for a total of three times. The tissue sections were kept dry to prevent antibody loss. Drop 10% goat serum into the drawn circles and incubate at room temperature for 30 min at room temperature. The blocking solution was removed and the following primary antibody was added and incubated overnight at 4°C: anti‐CD3 (Rab; Abcam; Cat.no.ab237721 ; 1:1000). The sections were subsequently placed flat in a wet box and incubated at 4°C overnight. The slides were placed in PBS (pH 7.4) and washed with a decolorization shaker for 5 min for three times. The HRP tag labeled with the corresponding species of the primary antibody (i. e., secondary antibody, SeraCare, Cat.no.5220‐0336,1:400) covered the tissue and incubated at room temperature for 50 min. The slides were placed in PBS (pH 7.4) and washed with a decolorization shaker for 5 min for three times. Tramine salt‐CY 3 was then added to the tissue (in PBST containing 0.003% hydrogen peroxide) and incubated for 20 min at room temperature. Repeated PBS washes three times, 5 min/time. Slides were put in one citric acid repair solution at high temperature for 6 min and naturally cooled to room temperature. After slightly drying the section, circle the tissue to prevent the loss of antibodies. Drop 10% goat serum into the drawn circle and incubate at room temperature.

Remove the blocking solution and drop the second primary antibody, anti‐CD8 (Rab; Abcam; Cat.no.ab217344; 1:1000), then the sections were placed flat in a wet box and incubated at 4°C overnight. The slides were placed in PBS (pH 7.4) and washed with a decolorization shaker for 5 min for three times. The HRP tag labeled with the corresponding species of the primary antibody (i.e., secondary antibody, SeraCare, Cat.no.5220‐0336,1:400) covered the tissue and incubated at room temperature for 50 min. The slides were placed in PBS (pH 7.4) and washed with a decolorization shaker for 5 min for three times. Tyramine salt‐488 was then added to the tissue (configured with PBST containing 0.003% hydrogen peroxide) and incubated for 20 min at room temperature. Repeated PBS washes three times, 5 min/time. Slides were put in one citric acid repair solution at high temperature for 6 min and naturally cooled to room temperature. After slightly drying the section, circle the tissue to prevent the loss of antibodies. Drop 10% goat serum into the drawn circle and incubate at room temperature.

**Figure 8:**
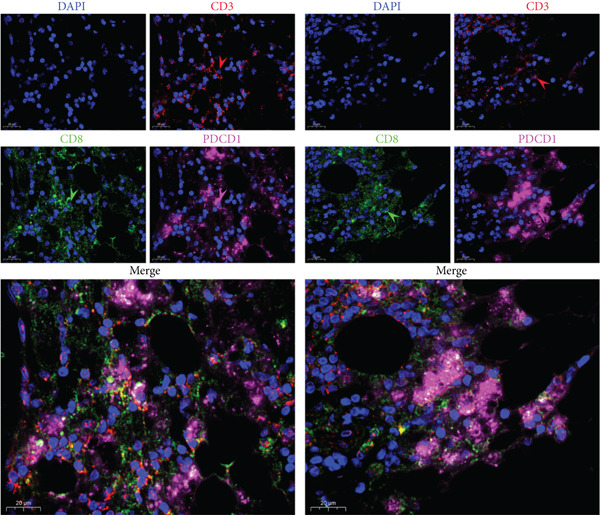
Expression of the depleted marker gene PDCD 1 in CD8+ T cells was verified by multiplex immunofluorescence, which indicates the presence of depleted CD8+ T cells in GCTB.UV‐excited nuclei are blue, corresponding to fluorescein‐labeled red/green/pink light shown as positive. Multiplex immunofluorescence confirmed the presence of PD CD 1 in the tumor tissue of GCTB + T cells (CD3+ CD8+), 20 *μ*m.

Remove the blocking solution and drop the third primary antibody, anti‐PDCD 1 (Rab; Abcam; Cat.no.ab237728 ; 1:500). The sections were subsequently placed flat in a wet box and incubated at 4°C overnight. The slides were placed in PBS (pH 7.4) and washed with a decolorization shaker for 5 min for three times. The HRP tag labeled with the corresponding species of the primary antibody (i.e., secondary antibody, SeraCare, Cat.no.5220‐0336,1:400) covered the tissue and incubated at room temperature for 50 min. The slides were placed in PBS (pH 7.4) and washed with a decolorization shaker for 5 min for three times. The currently available tyramine salt‐CY 5 (PBST containing 0.003% hydrogen peroxide) was then added to the tissue and incubated at room temperature for 20 min. Repeated PBS washes three times, 5 min/time. The slides were placed in PBS (pH 7.4) and washed with a decolorization shaker for 5 min for three times. Nuclei were counterstained with the DAPI dye solution and incubation for 10 min. The final slides were placed in PBS (pH 7.4) and washed for 5 min with a decoloring shaker for three times. Sections were sealed by anti‐fluorescence quenching of the blocking tablets. After sealing, the sections were observed under a fluorescence microscope and the images (Figure 8) were collected.

#### 3.1.8. Diversity of CD4+ T Cells in GCTB

We have classified 1265 reclustered CD4+ T cells into six distinct subgroups (Figure 9a). By employing an unbiased clustering method on the data and integrating information from literature and databases, we have characterized the following types of CD4+ T cells and their associated marker genes (Figure 9b): C1 (CCL20 and EGR1), C2 (IL7R), C3 (FOXP3, TIGIT, and IKZF2), C4 (GZMH and TNFSF9), C5 (CD40LG), C6 (CXCL13) (Figure 9b). Furthermore, we have visualized the overall plot of the expression levels of these marker genes for the six cell clusters using a bubble plot (Figure 9c).

Figure 9ScRNA‐seq profile of CD4+ T cells from human samples of GCTB. (a) UMAP plot of 1265 cells demonstrating the six main cell types in CD4+ T cells. (b) UMAP plots showing the expression of representative well‐known markers for each cell type in CD4+ T cells. (c) Bubble plots illustrate the expression levels of marker genes in CD4+ T cell clusters. (d) Proportional pie chart representing the proportions of six different cell types in CD4+ T cells samples.

**Figure:**
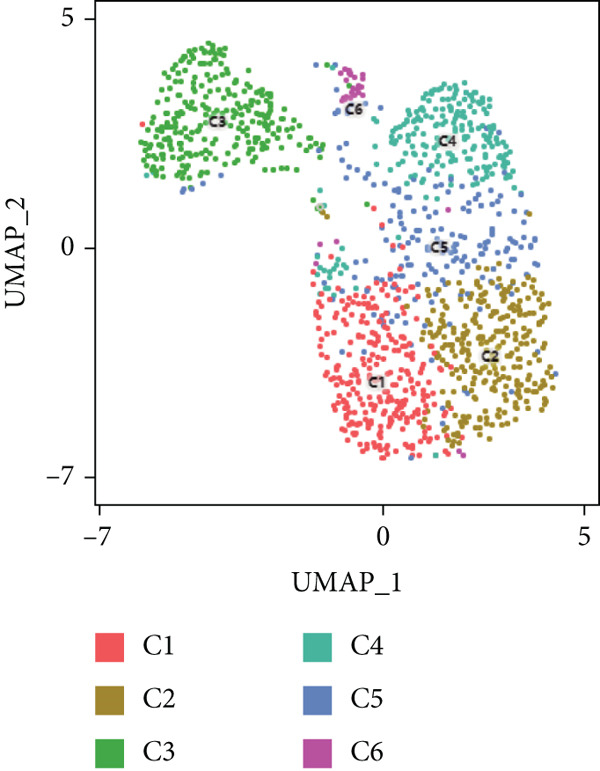
(a)

**Figure:**
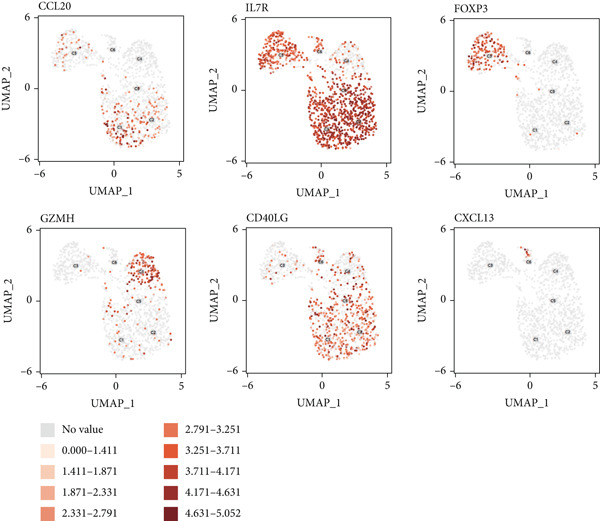
(b)

**Figure:**
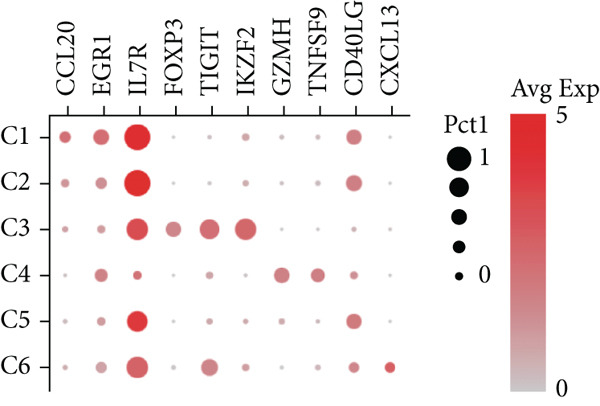
(c)

**Figure:**
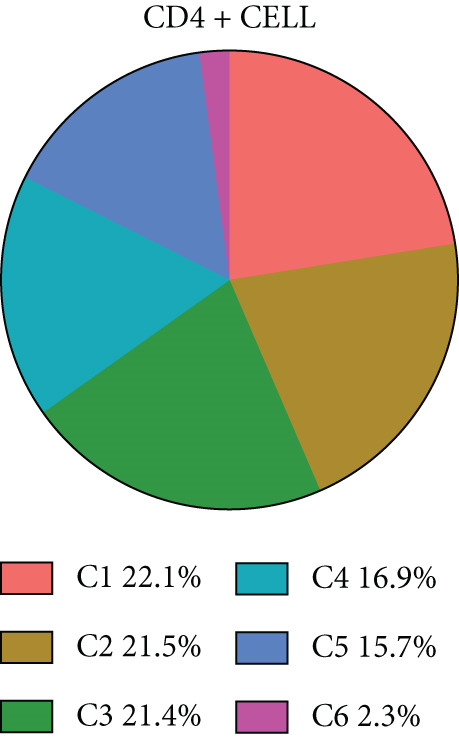
(d)

The abundance of CCL20 in C1 is consistent with earlier studies that have emphasized its ability to stimulate the development of granulocyte‐macrophage progenitors (GMPs) and facilitate the growth of immunosuppressive polymorphonuclear myeloid‐derived suppressor cells (PMN‐MDSCs) inside the TME. The involvement of the CCL20/CCR6 signaling pathway has been linked to immunosuppression in bone metastatic prostate cancer. Inhibiting this pathway could potentially ease T cell depletion and improve patient survival [49]. The significant enrichment of IL7R in C2 indicates the presence of a memory and residency phenotype. The presence of FOXP3, IKZF2, and TIGIT in C3 indicates that it belongs to a specific subtype of regulatory T cells (Tregs) [50]. Recent research suggests that the absence of IKZF2 in CD4+ T cells encourages the development of tissue‐invasive T cells and tissue inflammation [51]. On the other hand, TIGIT is regarded as one of the most promising targets for immune checkpoint therapy [52]. The significant abundance of GZMH and TNFSF9 in C4 indicates characteristics that are in line with activated CD4+ T cells. C5 demonstrates a significant concentration of CD40LG, mostly found in CD4+ helper subsets, such as follicular helper T cells (Tfh), Th1‐like, and Th subsets. CD40LG+ found in CD4+ helper cells that provide effective auxiliary signals can hinder the exhaustion program of CD8+ T cells [53]. A minority of C6 cells exhibit significant enrichment of CXCL13. Recent studies indicate that these cells have a vital role in the immune response to cancer in humans. T lymphocytes expressing CXCL13 have been shown to exert significant antitumor effects by interacting with DC [54, 55]. Our observation reveals that subtypes C1 and C3, which actively contribute to the development of tumors, are the predominant subtypes within CD4+ T cells (Figure 9d).

#### 3.1.9. Analysis of CD4+ T Cell Subtypes in GCTB Involves Studying Their Functional States and Differentiation Trajectory

To further examine the functional states and differentiation mechanisms of CD4+ T cells, we conducted GO enrichment analysis. This entailed categorizing highly expressed genes in CD4+ T cells into several functional categories, which allowed us to identify distinct BP for each subtype. Specifically, we found that subtype C1 is associated with protein refolding and cellular response to heat. Subtype C2 is linked to cytoplasmic translation and rRNA processing. Subtype C3 is involved in the T cell receptor signaling pathway, T cell homeostasis, and T cell activation. Subtype C4 is associated with immune system processes, adaptive immune response, and neutrophil chemotaxis. Subtype C5 is involved in the positive regulation of CD4‐positive, alpha‐beta T cell proliferation and T cell activation. Lastly, subtype C6 is associated with cell migration (Figure 10a).

Figure 10Characteristics and differentiation trajectory of CD4+ T cells in GCTB. (a) The bar chart depicts the study of gene ontology (GO) biological processes for six cell clusters of CD4+ T cells. (b) The bar chart displays the results of the Kyoto Encyclopedia of Genes and Genomes (KEGG) enrichment analysis for six cell clusters of CD4+ T cells. (c) The QuSAGE heat map displays the gene sets that are enriched in the six cell clusters of CD4+ T cells. (d) Single‐cell regulatory network inference and clustering (SCENIC) analysis revealed genes associated with the transcriptional regulatory network in different clusters of CD4+ T cells. (e) The Monocle trajectory analysis plot illustrates the progression of differentiation for the six clusters of CD4+ T cells.

**Figure:**
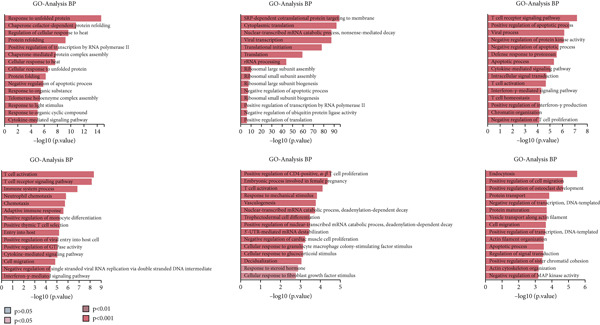
(a)

**Figure:**
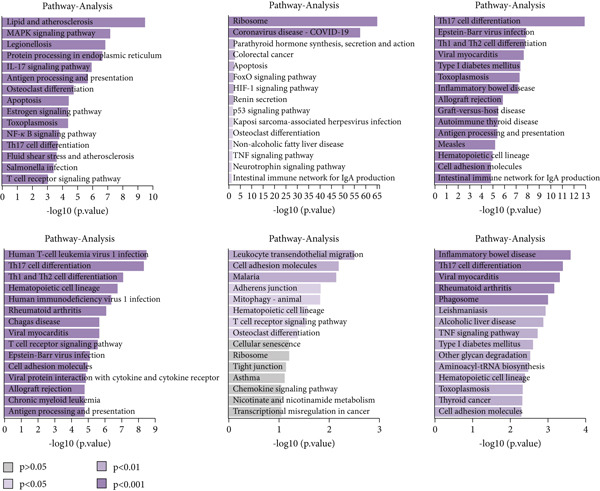
(b)

**Figure:**
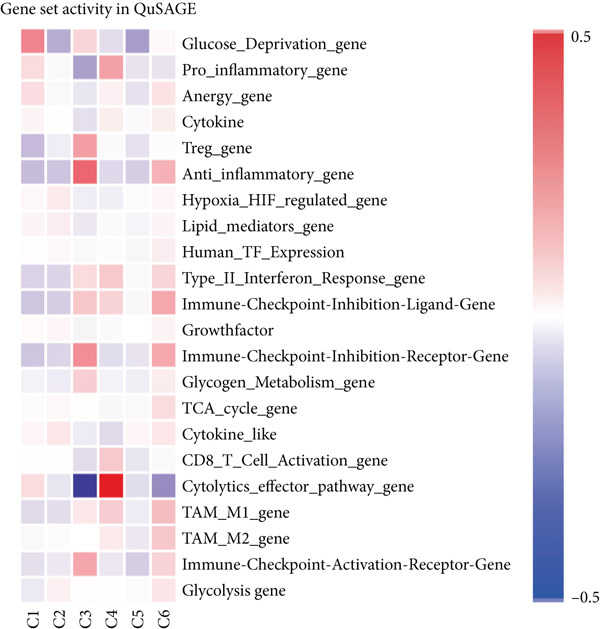
(c)

**Figure:**
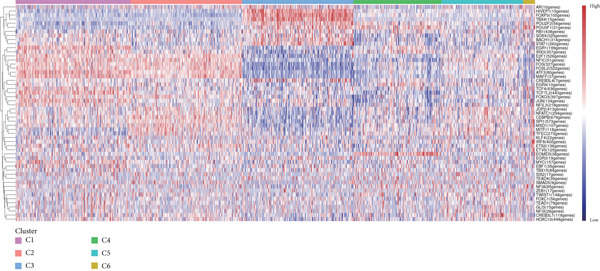
(d)

**Figure:**
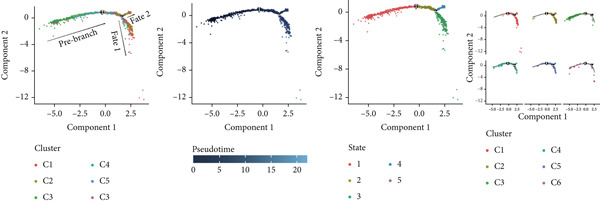
(e)

The KEGG enrichment analysis revealed pathways that showed varying degrees of enrichment across different cell groups. In cell group C1, the enriched pathways were Lipid and atherosclerosis, IL‐17 signaling pathway, and MAPK signaling pathway. In cell group C2, the enriched pathways were ribosome and COVID‐19. In cell group C3, the enriched pathways were Th17 cell differentiation, Th1 and Th2 cell differentiation. In cell group C4, the enriched pathways were Human T‐cell leukemia, Th17 cell differentiation, and Th1 and Th2 cell differentiation. In cell group C5, the enriched pathways were leukocyte transendothelial migration and cell adhesion molecules. In cell group C6, the enriched pathways were Th17 cell differentiation and TNF signaling pathway (Figure 10b).

The QuSAGE gene set analysis identified the gene sets that were enriched in the six subgroups of CD4+ T cells. The analysis revealed a higher enrichment of glucose deprivation genes in C1. C3 demonstrated significant enrichment in genes associated with anti‐inflammatory processes and regulatory T cells, while showing a negative connection with genes related to the cytolytic effector pathway. C4 exhibited an inverse pattern compared with C3 regarding the presence of enriched genes, indicating its capacity to augment immunological responses (Figure 10c).

The SCENIC analysis identified specific transcriptional regulatory network‐associated genes for different subtypes of CD4+ T cells (Figure 10d). Our results indicated a significant concentration of transcription factors in various CD4+ T cell clusters in GCTB exceeded the number of oncogenes and factors related to immune suppression [56]. By employing pseudo‐time trajectory analysis with the six cellular subtypes, we discovered (activated CD4+ T) cells. Meanwhile, the residency‐type C2 cells were mostly centered on the minor Fate2 branch. A limited number of C6 cells were scattered unevenly along the timeline, and the tumor immune suppressive C1 cells were found at the endpoints of both fate branches (Figure 10e). This illustrates the general immunosuppressive pattern of CD4+ T cells in GCTB.

### 3.2. Complex Cellular and Molecular Interaction Networks in GCTB

CellPhoneDB was used to examine intercellular communication in GCTB, we found important links between TAMs and cancer cells′ communication (Figure 11a). To demonstrate how TAMs interact with other cell clusters when functioning as receptors or ligands, respectively, we employed circos plots and bubble plots.(Figure 11b, 11c, 11d, and 11e) [57]. TAMs and T cells entails the stimulation of diverse immunological checkpoints, such as TIGIT, CD96, and TNFRSF14. This connection facilitates the promotion of immune evasion mechanisms during the progression of cancer [58]. The upregulation of CXCR3 and CCR5 suggests that the communication between TAMs and T cells has the potential to augment the malignancy of GCTB [59]. The RIPK1‐TNF pathway serves as the main means of communication between DC cells. RIPK1, a pivotal regulatory protein, governs the initiation of MAPK and NF‐*κ*B signaling pathways, resulting in cellular apoptosis, necrosis, and inflammation [60].

Figure 11Cell–cell communication network of CellPhoneDB in GCTB. (a) The heat map illustrates the number of protein–protein interaction pairs among the 10 cell clusters in GCTB. (b) The Circos plot depicts the precise pairs of protein–protein interactions between TAMs functioning as ligands and other clusters of cells. (c) The Bubble plot illustrates the potency and importance of protein–protein interactions between TAMs functioning as ligands and other cellular clusters. (d) The Circos plot depicts the precise pairs of protein–protein interactions between TAMs functioning as receptors and other clusters of cells. (e) The Bubble plot depicts the strength and significance of protein–protein interactions between TAMs acting as ligands and other cell clusters. (f) The Pair Network exhibits the precise protein–protein interaction pairs involving TAMs and tumor cells.

**Figure:**
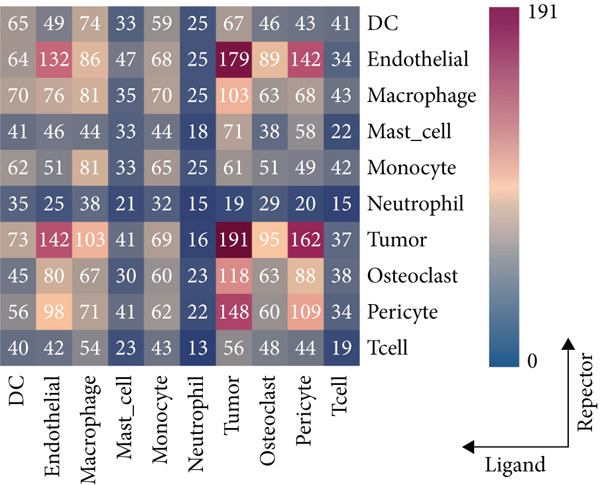
(a)

**Figure:**
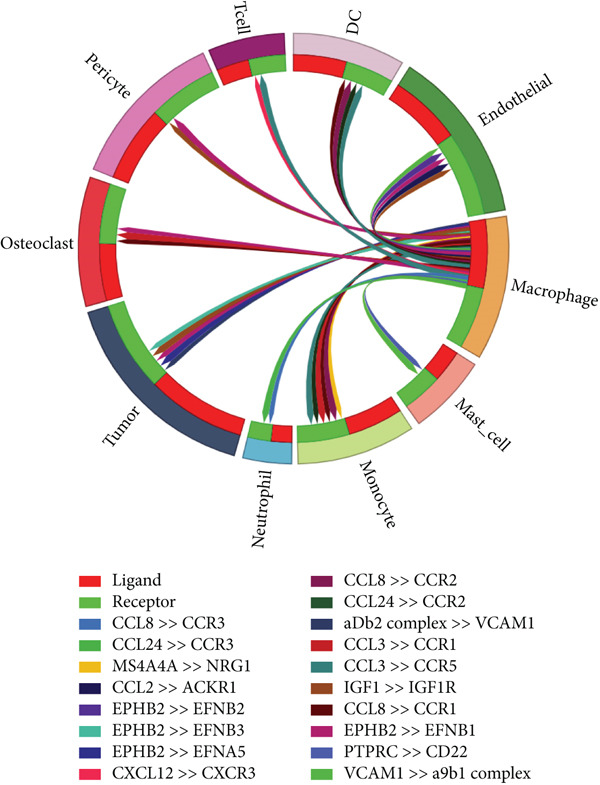
(b)

**Figure:**
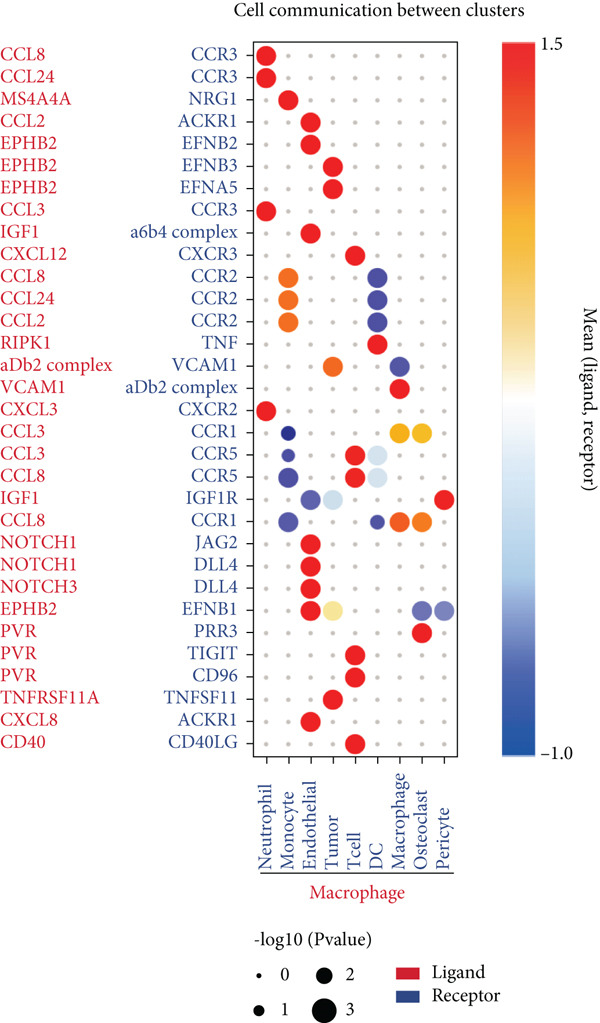
(c)

**Figure:**
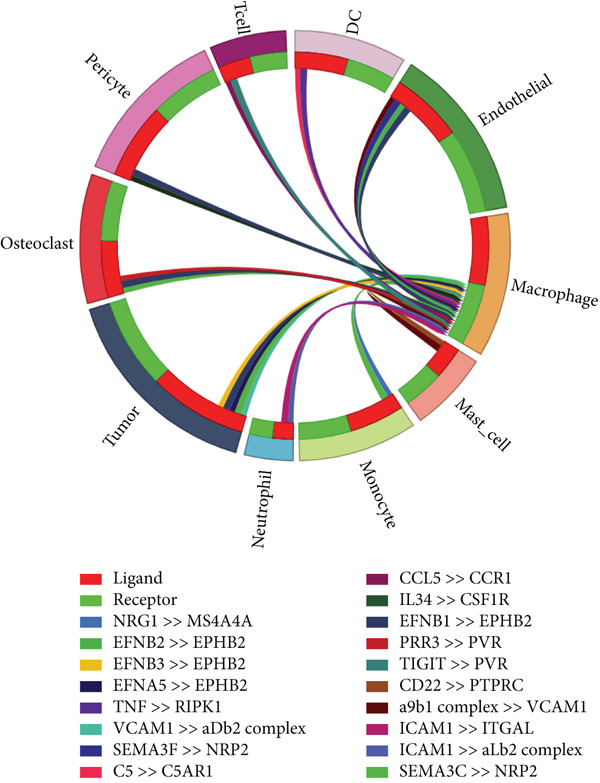
(d)

**Figure:**
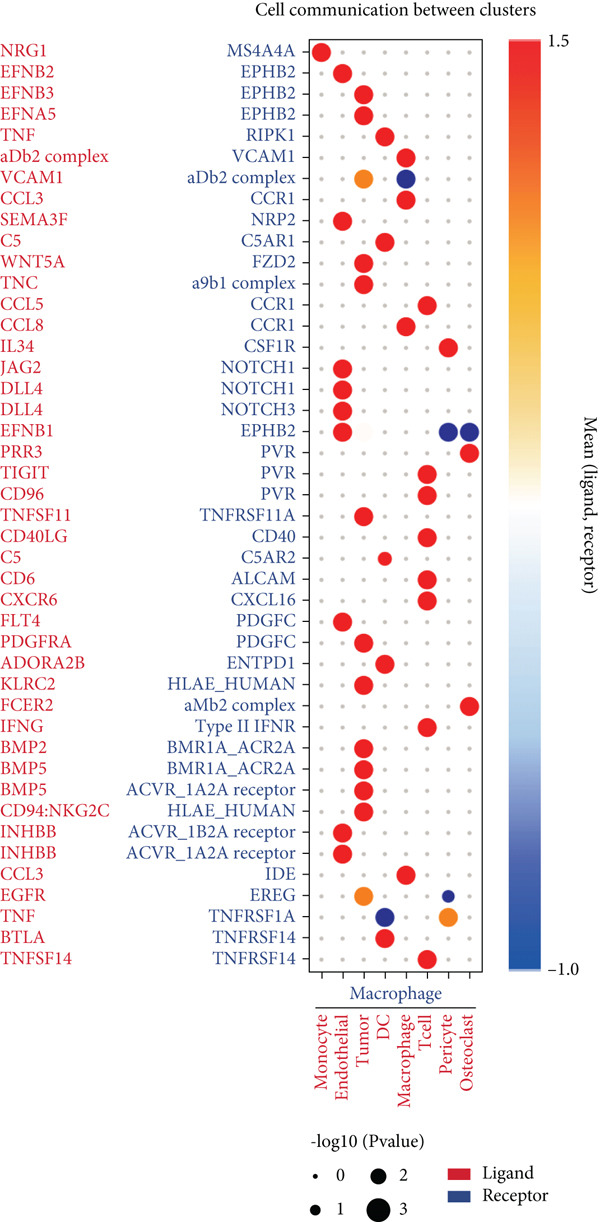
(e)

**Figure:**
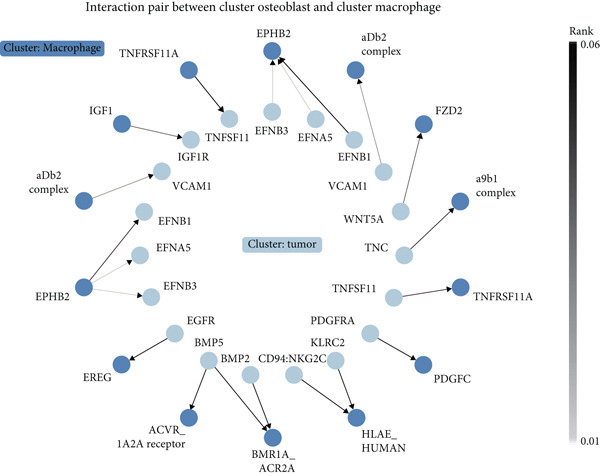
(f)

Furthermore, our research indicates that osteoclasts have the potential to attract TAMs via aMb2 [61]. The additional use of Pair Network highlights the notable importance of the TNFRSF11‐TNFRSF11A ligand‐receptor pair in the communication between TAMs and tumor cells (Figure 11f). GCTB arises because to the dynamic imbalance between osteoblast‐mediated bone matrix formation and osteoclast‐mediated bone resorption. Osteoblasts are stimulated by bone resorption to release receptor activator of nuclear factor‐*κ*B ligand (RANKL), which then binds to RANK on osteoclast precursors and multinucleated osteoclasts, stimulating the creation of multinucleated osteoclasts and initiating bone resorption [62]. Overexpression of RANKL leads to increased interaction between RANK and RANKL, which in turn stimulates the production of a large number of osteoclasts. The excessive activity of osteoclasts leads to substantial bone destruction and the secretion of extra cytokines that stimulate the growth of tumors. Ultimately, this process leads to the development of giant cell tumors [63]. Additionally, we noticed that EphB2 was actively communicating between tumor cells and macrophages and was substantially abundant in tumor cells. EphB2, a crucial member of the Eph receptor family, has been shown to exhibit aberrant expression in hepatocellular carcinoma, colorectal cancer, gastric cancer, and other cancers, resulting in the development of tumors. We hypothesize that it also plays a part in the development of GCTB [64].

## 4. Discussion

This study performed an exploratory single‐cell transcriptomic analysis of GCTB, providing an initial depiction of the cellular landscape and intercellular communication within the TME. Ten cellular subtypes were identified, and their distinguishing gene signatures were defined, providing initial insights into the immunological heterogeneity of GCTB. Among these, TAMs emerged as the dominant infiltrating immune population [65].

An extensive investigation into TAMs yielded valuable insights into their differentiation mechanisms and roles in GCTB. Macrophages have been associated with facilitating the onset and advancement of cancer by stimulating angiogenesis and assisting in the migration of tumor cells. TAMs are potential candidates for anticancer treatment, either by eliminating them or inducing their transformation into anti‐tumoral states [66]. Our analysis may suggest additional roles of TAMs in GCTB immune regulation. Pseudo‐time trajectories further indicated potential differentiation pathways of TAMs, consistent with the existence of diverse functional subtypes, which may contribute differently to immune modulation and tumor progression.

CD8+ T cells displayed heterogeneous states of activation and exhaustion, consistent with immunosuppressive features of the GCTB microenvironment. The presence of subsets enriched in immune checkpoint–related genes suggests challenges for immune checkpoint blockade therapy in this disease [67]. The utilization of pseudo‐time trajectory analysis yielded valuable insights on the dynamic reactions of CD8+ T cells, as they transitioned from stages of proliferation to levels of cytotoxicity and fatigue. T cell exhaustion is a significant impediment to the effectiveness of antitumor immune responses. Nevertheless, the exact role of other immune cells in the TME in causing this functional imbalance is still unknown [68]. Research has demonstrated significant associations between TAMs and exhausted T cells (Tex) in the TME [69].

An extensive examination of CD4+ T cells unveiled the diverse variances and functional roles of these cells in GCTB. The existence of many subtypes, particularly the abundance of Tregs, underscores the immunosuppressive characteristics of the immunological milieu in GCTB [70], facilitating immune evasion within tumors. The process of differentiation of CD4+ T cells was demonstrated by pseudo‐time trajectory analysis.

The CellPhoneDB analysis identified potential ligand‐receptor interactions between TAMs and tumor cells, encompassing pathways such as RANKL–RANK and EPHB2–EFNB1. These interactions may elucidate processes of immune modulation and bone remodeling in GCTB, though validation in larger cohorts is required [71]. The findings align with previous research on the RANKL pathway while also pointing toward unexplored immune‐mediated processes that warrant future study [72].

Taken together, this study provides preliminary insights into the immune microenvironment of GCTB at single‐cell resolution. However, several limitations must be emphasized. First, the analysis was based on a single patient sample, which limits generalizability and should be considered hypothesis‐generating rather than definitive. Second, the technical constraints of scRNA‐seq, including dropout effects, potential doublets, and incomplete capture of transcriptomes, may have influenced the results. Finally, the absence of functional or clinical validation restricts the interpretation of predicted cell states and interactions.

Despite these limitations, this preliminary study lays the groundwork for subsequent research endeavors. Validation in larger patient cohorts, combined with functional assays and integration of clinical data, will be essential to confirm the cellular phenotypes, interaction networks, and therapeutic implications suggested by this study. By characterizing the transcriptomic diversity of GCTB at single‐cell resolution, this pilot study offers a framework for future research into immune regulation and targeted therapies in this rare tumor.

There are multiple constraints included in this investigation. Initially, the sample size is limited and may not encompass the complete diversity of GCTB. Furthermore, limitations in the process of isolating and capturing individual cells, along with the inability to guarantee the preservation of cell membranes′ integrity and functionality, may result in the distortion of cells′ authentic in vivo state during future analysis. Ultimately, while we conducted preliminary investigations on cell–cell interactions, the specific mechanisms require more experimental confirmation.

Ultimately, this study provides a novel perspective on the immunological composition of GCTB through a comprehensive analysis of gene expression in individual cells. The identified cellular phenotypes, physiological states, and intercellular communication networks lay the groundwork for future research and the development of targeted therapeutic strategies.

## Ethics Statement

The studies involving humans were approved by the Ethics Committee of Guangzhou Red Cross Hospital (Approval number 2023‐377‐01). The studies were conducted in accordance with the local legislation and institutional requirements. The participants provided their written informed consent to participate in this study.

## Disclosure

All authors read and approved the final manuscript.

## Conflicts of Interest

The authors declare no conflicts of interest.

## Author Contributions

Yiming Liu, Wei Luo, and Yude Xu contributed equally to this work and share first authorship. Yiming Liu, Wei Luo and Yude Xu designed the study and wrote the manuscript. Xiguan Yao acquired the data. Libing Dai, Qiao Feng, Peigeng Wang, Weichao Yang, and Yi Feng contributed significantly to the analysis of data. Haixiong Miao, Suixiang Huang and Dongping Ye revised the work critically for important intellectual content.

## Funding

This work was supported by the Guang Dong Basic and Applied Basic Research Foundation (2019A1515110723 to Dongping Ye); the Guangzhou Science and Technology Planning Project (202102010111 to Dongping Ye and 2023A03J0574 to Suixiang Huang); the Guangzhou Medical and Health Project (20201A011021 to Dongping Ye); the Guangdong Science and Technology Planning Project (B2019064 to Dongping Ye); and Guangzhou Key Disciplines in Medical Sciences Project (2025‐2027).

## Data Availability

The data that support the findings of this study are openly available in Gene Expression Omnibus at https://www.ncbi.nlm.nih.gov/geo/, reference number GSE254672.
